# The association between visceral adiposity index and risk of type 2 diabetes mellitus

**DOI:** 10.1038/s41598-024-67430-x

**Published:** 2024-07-18

**Authors:** Haoran Zhou, Tianshu Li, Jie Li, Xin Zhuang, Jie Yang

**Affiliations:** 1 Shandong University of Traditional Chinese Medicine, Jinan, China; 2 Department of Cardiology, The Affiliated Hospital of Shandong University of Traditional Chinese Medicine, Jinan, 250000 China

**Keywords:** T2DM_1_, VAI_2_, NHANES_3_, Cross-sectional study_4_, Relationship_5_, Obesity, Epidemiology

## Abstract

Relationships between the visceral adiposity index (VAI) and type 2 diabetes mellitus (T2DM) have been underexplored. The purpose of this study is to explore association between VAI and T2DM in adults in the US. Based on the National Health and Nutrition Examination Survey 2007–2018, 11,214 participants aged 20 years or older were included in a cross-sectional study. Multifactorial logistic regression analysis and smoothed curve fitting analysis were performed to investigate links between VAI and the prevalence of T2DM, as well as the stability and incidence between subgroups. In a fully adjusted continuous model, the aggregate population risk of T2DM increased 0.43-fold with each 1-unit increase in VAI [odds ratio (OR) = 1.43; 95% confidence interval (CI) 1.35–1.50]. In the fully adjusted categorical model with VAI scores stratified by quartiles, results showed a higher T2DM advantage among participants in the second, third, and fourth quartiles (Q2: OR 1.35, 95% CI 1.06–1.71; Q3: OR 2.46, 95% CI 1.95–3.11; Q4: OR 4.42, 95% CI 3.55–05.50). Compared with Q1, the prevalence of T2DM in the total population increased 3.42-fold in Q4. The above results indicated that VAI was positively associated with the prevalence of T2DM, which was consistent and nonlinear with the smoothed curve-fitting analysis (P for non-linear = 0). Subgroup analyses after adjusting for covariates showed that keeping with the overall population results, it also was found that there was an interaction between sex and hypertension in the subgroups. VAI was positively associated with the prevalence of T2DM and was more prevalent in women, non-hypertensive than in men, hypertensive populations.

## Introduction

Diabetes mellitus (DM) is a metabolic disease with multiple risk factors. Persons with DM are prone to various complications, and the disease thus poses a serious threat to human health. According to the International Diabetes Federation, in 2021, approximately 537 million people globally had been diagnosed with DM, and its prevalence continues to grow: the number of persons with DM is projected to grow to 643 million by 2030 and 783 million by 2045^1^. The type 2 diabetes mellitus (T2DM) is the most predominant type of DM, as it accounts for more than 90% of all patients with DM^2,3^, and it has been estimated that there will be approximately 439 million people with T2DM by 2030^4^. Such an alarming growth rate has important implications for the global health system, and curbing the global growth of T2DM would be an important step toward reducing economic burdens and improving people’s health and well-being^5^. Obesity has been found to be a crucial contributor to the development of T2DM. Obesity is typically a chronic, relapsing, and multifactorial disease that affects every organ system and that often leads to metabolic disorders or other related comorbidities that affect physical and mental health^6^. The risk of T2DM has been found to be approximately sevenfold higher in persons defined as obese (body mass index (BMI) > 30) than in persons who are not considered to be overweight (BMI < 25)^7^, and large amounts of visceral fat deposits and ectopic fat have been shown to be previously underappreciated risk factors for T2DM^8^. Accordingly, in a study employing multiparametric magnetic resonance imaging (MRI), larger amounts of visceral adipose tissue were shown to correlate with a higher risk of developing T2DM^9^.

While MRI and computed tomography (CT) are clinically accurate and reliable in the assessment of visceral fat, these techniques are complex, require expensive equipment, and may be associated with adverse side effects^10^. Therefore, there remains a need for a more convenient, practical, user-friendly, and safe method to assess visceral fat. In this regard, Marco et al. proposed a new index, the Visceral Adiposity Index (VAI), for the evaluation of visceral fat; this assessment tool is based on the results of conventional biochemical tests quantifying blood triglyceride (TG) and high-density lipoprotein (HDL) levels as well as the anthropometric indices BMI and waist circumference index (WC)^11^. The VAI is regarded as a surrogate measure of the visceral fat profile and visceral fat dysfunction that performs with higher sensitivity and specificity than do classical parameters^12^. The results of VAI analyses have been widely used to study heart failure, hyperuricemia, cardiovascular and cerebrovascular diseases, stroke, and other diseases^13–16^, and recent studies have demonstrated a non-linear association between VAI and fasting glucose levels and indexes of renal function^7,10^.

In order to perform a comprehensive analysis of the use of VAI in the clinical diagnosis of T2DM, the present study employed information from a large and representative source of data: the National Health and Nutrition Examination Survey (NHANES) from the United States National Centers for Disease Control and Prevention. We used information from this study to probe associations between VAI and T2DM, providing a clinical tool for early risk assessment, prevention, diagnosis, and treatment.

## Materials and methods

### Study population

**Figure 1:**
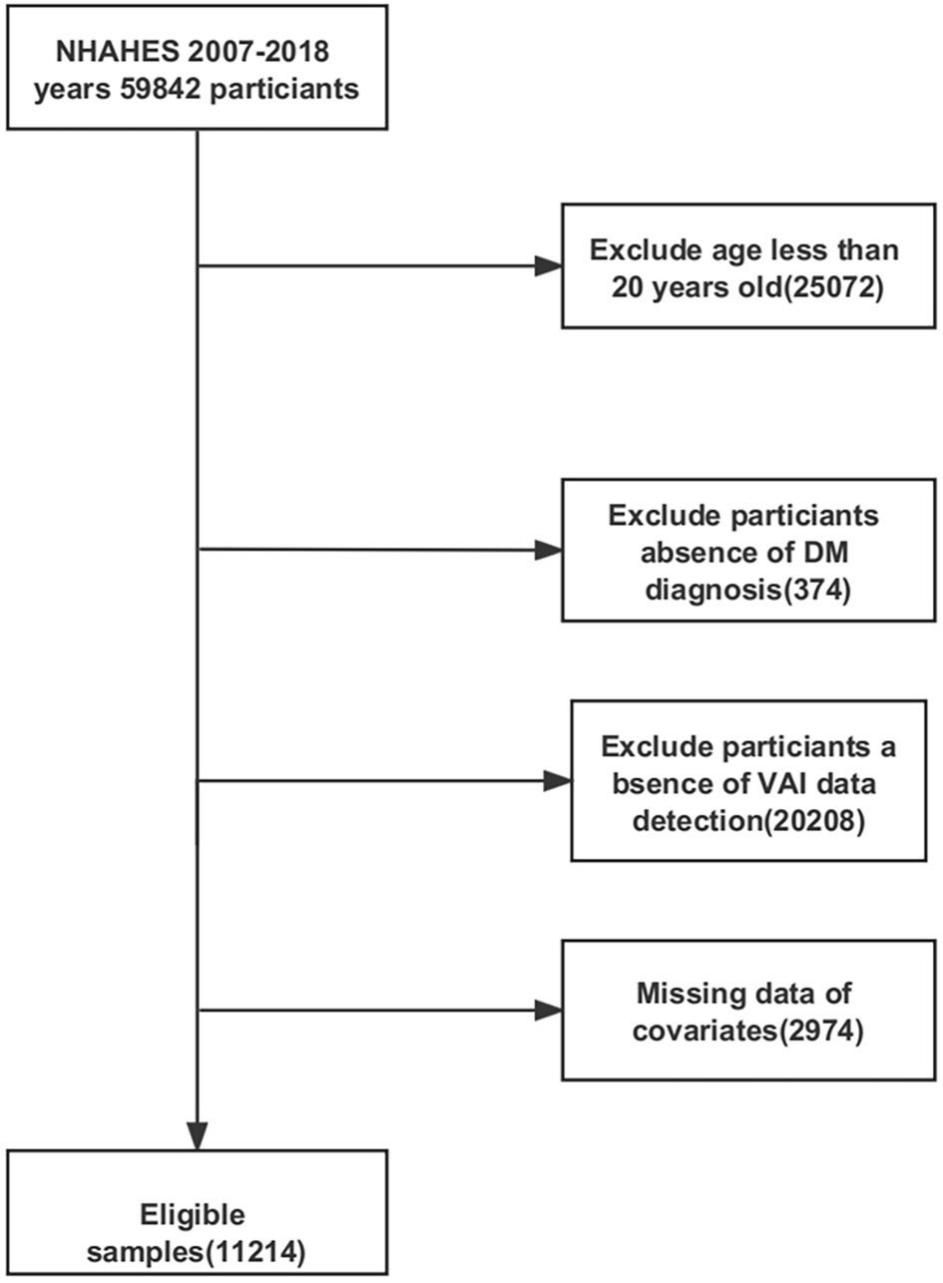
Flowchart of the study population from NHANES 2007–2018.

NHANES is a series of large-sample, representative, cross-sectional surveys that aims to understand the health status of citizens of the US by focusing primarily on diet^17^. The types of data available include demographic data, dietary data, physical measurements, laboratory data, and questionnaire data. The data from the six cycles have been standardized. For the present study, a total of 59,842 participants were extracted from the NHANES database, and data were ultimately used from 11,214 participants. Exclusion criteria included the following factors: 25,072 subjects were aged less than 20 years; Data missing for 374 participants with T2DM; for 20,208 subjects, complete data to calculate the VAI index were not available; and 2974 were missing data on covariates (Fig. 1).

### Data collection

Information was extracted and recorded by specialized personnel. The information extracted included age, gender, ethnics, education, marriage status, Income to poverty ratio (PIR), BMI, WC, smoking status, and alcohol consumption, estimated glomerular filtration rate (eGFR), physical activity, as well as data regarding typical biochemical markers, such as blood TG levels. The information also included self-reported answers to questionnaires related to hypertension, coronary heart disease, stroke, and heart failure. As part of the original survey, relevant personnel collated the basic information, and the biochemical samples were stored appropriately prior to submission to laboratories of the University of Minnesota and the University of Missouri-Columbia for examination and analysis.

### Diagnosis of type 2 diabetes

A diagnosis of T2DM was made upon the fulfilling any one of the below criteria: (1) self-reported physician diagnosis of T2DM; (2) current use of glucose-lowering medications or insulin injections; (3) random blood glucose level of at least 11.1 mmol/L; (4) glycosylated hemoglobin (HbA1c) level of at least 6.5%; (5) fasting blood glucose (FPG) level of at least 7.0 mmol/L; or (6) 2-h oral glucose tolerance test glucose level of at least 11.1 mmol/L.

### Measurement of the visceral adiposity index

VAI estimates visceral fat using anthropometric data (BMI and WC) together with metabolic parameters (TG and HDL-C levels, in units of mmol/L). Higher VAI values correlate with more visceral fat. The formulas used for male and female subjects are shown below as equations (1) and (2), respectively. (1) VAI = (WC/[39.68 + 1.88 × BMI)] × (TG/1.03) × (1.31/HDL-C); (2) VAI = [WC/(36.58 + 1.89 × BMI)] × (TG/0.81) × (1.52/HDL-C). In the present study, VAI was analyzed as a continuous variable, and participants were grouped according to VAI quartiles.

### Selection of covariates

The main covariates considered included the demographic factors age; sex (male or female); race (Mexican–American, non-Hispanic white, non-Hispanic black, other Hispanic, and other races); educational attainment (less than 9th grade, 9th–11th grade, high school diploma, some college, or completion of college and above); and marriage status (married, widowed, divorced, separated, unmarried, or cohabitating); PIR (< 1.3, 1.3–3.5, ≥ 3.5). They also included the health-related behaviors smoking (former, current, or never) and alcohol consumption (former, light, heavy, or non-drinking), physical activity. The laboratory markers included: total cholesterol (TC), low-density lipoproteins (LDL), fasting plasma glucose (FPG), glycosylated hemoglobin (HbA1c), estimated glomerular filtration rate (eGFR), alanine aminotransferase (ALT), and aspartate aminotransferase (AST). Common underlying diseases were also included. These diseases included hypertension, which was recognized as satisfying any one of these criteria: self-reported physician diagnosis of hypertension; current usage of BP-lowering drugs, or average of 3 measurements of blood pressure with systolic blood pressure (SBP) ≥ 140 mmHg and/or diastolic blood pressure (DBP) ≥ 90 mmHg. They also included coronary heart disease (CHD), stroke, and heart failure; each of these diseases was monitored according to self-reporting of a physician’s diagnosis. Specific technical methods regarding covariate determination are available from the official NHANES website.

### Statistical analysis

The survey data were analyzed using R (version 4.3.1). Continuous variables are expressed as mean ± standard deviation, and categorical variables are expressed as frequencies (percentages). Differences between categorical variables were analyzed using the chi-square test, and differences between continuous variables were assessed using the weighted Student’s t-test test or the Mann–Whitney U-test, depending on the nature of the distribution. The level of statistical significance was set at *P* < 0.05. The association between VAI and T2DM in adults from the US was analyzed by building three multivariate logistic regression models. Model 1 was not adjusted for confounders; model 2 was adjusted for age, gender, and ethnicity; model 3 was further adjusted for education, marriage status, PIR, smoking, and alcohol consumption, physical activity, eGFR, AST, AST, TC, LDL, hypertension, coronary heart disease, stroke, and heart failure. Smoothed curve fitting was then used to explore whether there was a non-linear relationship between VAI and T2DM. Finally, the confounders listed in the baseline table age (20–34, 35–54, 55–74, ≥ 75), sex (male or female), race (Mexican–American, non-Hispanic white, non-Hispanic black, other Hispanic, and other races), smoking, alcohol consumption, eGFR (< 90, ≥ 90), CHD (yes/no), stroke (yes/no), and heart failure (yes/no) were analyzed using stratified logistic regression modeling in a subgroup analysis for the presence of interactions.

### Ethics approval and consent to participate

All participants signed informed consent forms, and the NHANES program was approved by the Ethics Review Board of the US National Center for Health Statistics. The present study adhered to the guidelines outlined in the Epidemiologic Statement for Enhanced Reporting of Observational Studies^18^.

### Ethics statement

The studies involving human participants were reviewed and approved by The Research Ethics Review Board at the National Center for Health Statistics (NCHS). The patients/participants provided their written informed consent to participate in this study.

## Results

### Baseline characteristics

The final inclusion of 11,214 patients in this study is shown in Fig. 1. The baseline characteristics by different disease states are shown in Table 1. The weighted mean age of the participants was 47.65 ± 0.29 years. Among the study participants, 49.00% were male, and 51.00% were female. VAI values were higher in diabetic patients than in non-diabetic patients. Supplementary Table S1, the participants were divided into four groups based on VAI quartiles; the values of VAI in these quartiles (as mean ± SD) were Q1, 0.59 ± 0.00; Q2, 1.10 ± 0.00; Q3, 1.79 ± 0.01; and Q4, 3.74 ± 0.04. Compared with subjects in Q1, subjects in Q4 were higher in age and more likely to be female and to belong to the non-Hispanic white demographic. As compared with subjects in Q1, subjects in Q4 had a higher average level of education, were more likely to be married, were more likely to be non-smokers and to not consume alcohol. In addition, subjects in Q4 had higher levels of BMI, WC, PIR, FDG, HbA1c, TC, TG, LDL, ALT, and AST, and higher levels of hypertension, coronary heart disease, stroke, and heart failure. As compared with subjects in Q1, subjects in Q4 were less likely to belong to the physical activity, and they had lower levels of eGFR and HDL. Differences in age, race, education, marriage, PIR, smoking, alcohol consumption, BMI, WC, FDG, HbA1c, TC, TG, LDL, HDL, eGFR, AST, AST, physical activity, hypertension, coronary artery disease and heart failure were statistically significant among all four quartiles (*P* < 0.05).

**Table 1:** Characteristics of study population divided by different disease states (n = 11,214).

Variable	Total (n = 11,214)	NoneT2DM (n = 8881)	T2DM (n = 2333)	*P* value
Age, years	47.65 ± 0.29	45.47 ± 0.31	59.21 ± 0.38	< 0.0001
Gender, n (%)				0.35
Female	5657 (50.60)	4545 (50.81)	1112 (49.46)	
Male	5557 (49.40)	4336 (49.19)	1221 (50.54)	
Race, n (%)				0.001
Mexican American	1655 (8.08)	1268 (7.96)	387 (8.76)	
Non-Hispanic Black	2192 (9.76)	1663 (9.28)	529 (12.29)	
Non-Hispanic White	4966 (69.69)	4043 (70.30)	923 (66.42)	
Other Race	1252 (7.11)	1034 (7.17)	218 (6.78)	
Others	1149 (5.37)	873 (5.29)	276 (5.75)	
Education, n (%)				< 0.0001
9–11th grade	1553 (10.20)	1159 (9.65)	394 (13.10)	
College graduate or above	2791 (31.56)	2394 (33.30)	397 (22.26)	
High school graduate	2545 (22.60)	1977 (21.83)	568 (26.70)	
Less than 9th grade	1015 (4.63)	664 (3.97)	351 (8.14)	
Some College	3310 (31.02)	2687 (31.25)	623 (29.81)	
Marital status, n (%)				< 0.0001
Divorced	1253 (10.61)	952 (10.28)	301 (12.36)	
Living with partner	917 (8.08)	815 (8.69)	102 (4.86)	
Married	5831 (56.23)	4503 (55.36)	1328 (60.85)	
Never married	2018 (17.68)	1805 (19.26)	213 (9.27)	
Separated	371 (2.12)	285 (2.08)	86 (2.31)	
Widowed	824 (5.28)	521 (4.33)	303 (10.35)	
PIR				< 0.0001
< 1.30	3450 (20.75)	2689 (20.31)	761 (23.06)	
1.30–3.50	4262 (35.48)	3295 (34.74)	967 (39.43)	
≥ 3.50	3502 (43.77)	2897 (44.95)	605 (37.51)	
eGFR, mL/min/1.73 m^2^	94.92 ± 0.37	96.83 ± 0.41	84.73 ± 0.61	< 0.0001
BMI, kg/m^2^	28.97 ± 0.11	28.28 ± 0.11	32.67 ± 0.21	< 0.0001
WC, cm	99.36 ± 0.27	97.27 ± 0.28	110.50 ± 0.49	< 0.0001
FPG, mmol/L	5.90 ± 0.02	5.48 ± 0.01	8.17 ± 0.09	< 0.0001
HbA1c, %	5.62 ± 0.01	5.38 ± 0.01	6.91 ± 0.04	< 0.0001
Physical Activity, METs	3853.99 ± 93.58	4137.17 ± 105.74	2347.98 ± 117.44	< 0.0001
TC, mmol/L	4.98 ± 0.02	5.02 ± 0.02	4.77 ± 0.03	< 0.0001
TG, mmol/L	1.37 ± 0.01	1.30 ± 0.01	1.71 ± 0.03	< 0.0001
HDL, mmol/L	1.41 ± 0.01	1.43 ± 0.01	1.28 ± 0.01	< 0.0001
LDL, mmol/L	2.95 ± 0.01	2.99 ± 0.01	2.75 ± 0.03	< 0.0001
Alt, U/L	25.10 ± 0.21	24.63 ± 0.23	27.56 ± 0.43	< 0.0001
Ast, U/L	25.08 ± 0.19	24.81 ± 0.21	26.53 ± 0.44	< 0.001
VAI	1.80 ± 0.02	1.66 ± 0.02	2.52 ± 0.07	< 0.0001
Smoking status, n (%)				< 0.0001
Former	2773 (25.70)	2007 (24.13)	766 (34.08)	
Never	6179 (55.28)	4998 (56.24)	1181 (50.13)	
Now	2262 (19.02)	1876 (19.63)	386 (15.79)	
Alcohol use, n (%)				< 0.0001
Former	1713 (12.51)	1163 (11.04)	550 (20.30)	
Heavy	2293 (21.05)	1960 (22.35)	333 (14.15)	
Mild	3958 (38.46)	3161 (38.69)	797 (37.26)	
Moderate	1757 (17.83)	1499 (18.67)	258 (13.37)	
Never	1493 (10.15)	1098 (9.26)	395 (14.92)	
Hypertension, n (%)				< 0.0001
No	6492 (62.23)	5792 (68.05)	700 (31.27)	
Yes	4722 (37.77)	3089 (31.95)	1633 (68.73)	
Stroke, n (%)				< 0.0001
No	10,785 (97.10)	8628 (97.77)	2157 (93.53)	
Yes	429 (2.90)	253 (2.23)	176 (6.47)	
CHD, n (%)				< 0.0001
No	10,766 (96.52)	8652 (97.80)	2114 (89.74)	
Yes	448 (3.48)	229 (2.20)	219 (10.26)	
HF, n (%)				< 0.0001
No	10,889 (97.74)	8722 (98.59)	2167 (93.23)	
Yes	325 (2.26)	159 (1.41)	166 (6.77)	

PIR, Income to poverty ratio; eGFR, estimated glomerular filtration rate; BMI, body mass index; WC, waist circumference; FPG, fasting plasma glucose; HbA1c, hemoglobin A1c; TC, total cholesterol; TG, triglyceride; HDL, high-density lipoprotein; LDL-c, low-density lipoprotein-cholesterol; ALT, alanine aminotransferase; AST, aspartate aminotransferase; CHD, coronary heart disease; HF, heart failure; DM, diabetes mellitus.

### Association between VAI and T2DM

**Figure 2:**
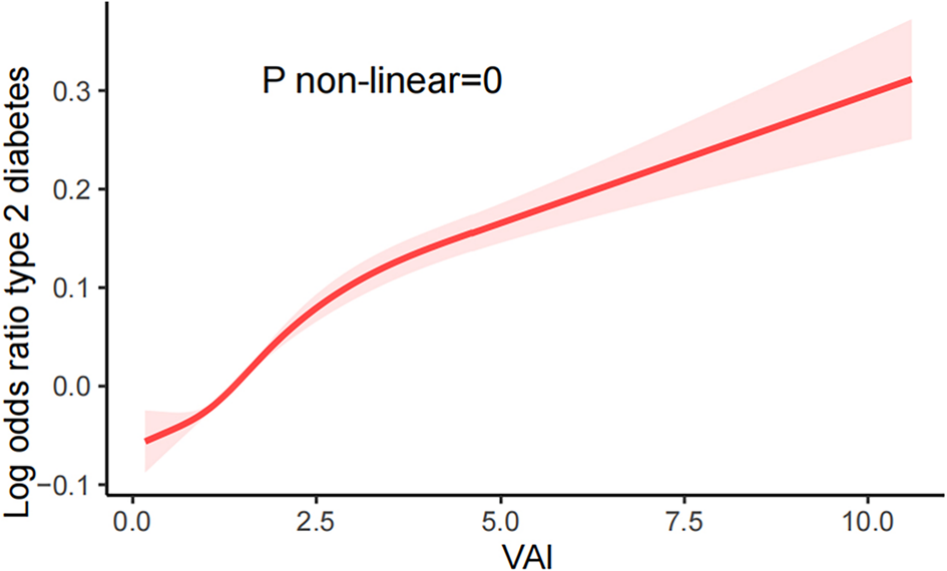
Curve fitting analysis of VIA and T2DM.

Logistic regression models were used to analyze the association among VAI and the prevalence of T2DM as shown in Table 2. In fully adjusted continuous models, the prevalence of T2DM in the total population increased 0.43-fold with each 1-unit increase in VAI [(OR) = 1.43; 95% (CI) 1.35–1.50]. In the fully adjusted categorical model with VAI scores stratified by quartiles, the results showed a higher T2DM advantage for participants in the second, third, and fourth quartiles (Q2: OR 1.35, 95% CI 1.06–1.71; Q3: OR 2.46, 95% CI 1.95–3.11; Q4: OR 4.42, 95% CI 3.55–05.50). The prevalence of T2DM in the total population increased 3.42-fold in Q4 compared to Q1. A trend test was performed to ensure the stability of the results of this study, and the incidence of VAI and T2DM showed a monotonically increasing trend in all models (*P* < 0.0001). In summary, it can be seen that VAI was positively correlated with the incidence of T2DM. We plotted a smoothed curve fit as shown in Fig. 2, and after adjusting for confounders such as age, sex, race, education, marriage, PIR, smoking, alcohol consumption, physical activity, eGFR, AST, AST, TC, LDL, hypertension, coronary heart disease, stroke, and heart failure, we found that VAI and T2DM prevalence were consistent and nonlinearly associated with multifactorial logistic regression (P for non-near = 0 < 0.05).

**Table 2:** The association between VAI and T2DM in a multiple logistics regression model.

Character	Model 1		Model 2		Model 3	
	95% CI	*P*	95% CI	*P*	95% CI	*P*
VAI	1.40 (1.35, 1.45)	< 0.0001	1.44 (1.38, 1.50)	< 0.0001	1.43 (1.35, 1.50)	< 0.0001
VAI group						
Q1	1 (ref)		1 (ref)		1 (ref)	
Q2	1.50 (1.20, 1.87)	< 0.001	1.44 (1.14, 1.82)	0.003	1.35 (1.06, 1.71)	0.02
Q3	2.84 (2.39, 3.37)	< 0.0001	2.72 (2.23, 3.32)	< 0.0001	2.46 (1.95, 3.11)	< 0.0001
Q4	4.76 (4.03, 5.61)	< 0.0001	4.89 (4.04, 5.93)	< 0.0001	4.42 (3.55, 5.50)	< 0.0001
p for trend		< 0.0001		< 0.0001		< 0.0001

Model 1: non-adjusted.

Model 2: adjutesd age, gender, race.

Model 3: adjutesd age, gender, race, education, marital, PIR, smoke, alcohol, eGFR, TC, LDL, Alt, Ast, Physical Activity, Hypertension, stroke, CHD, HF.

### Subgroup analysis

**Figure 3:**
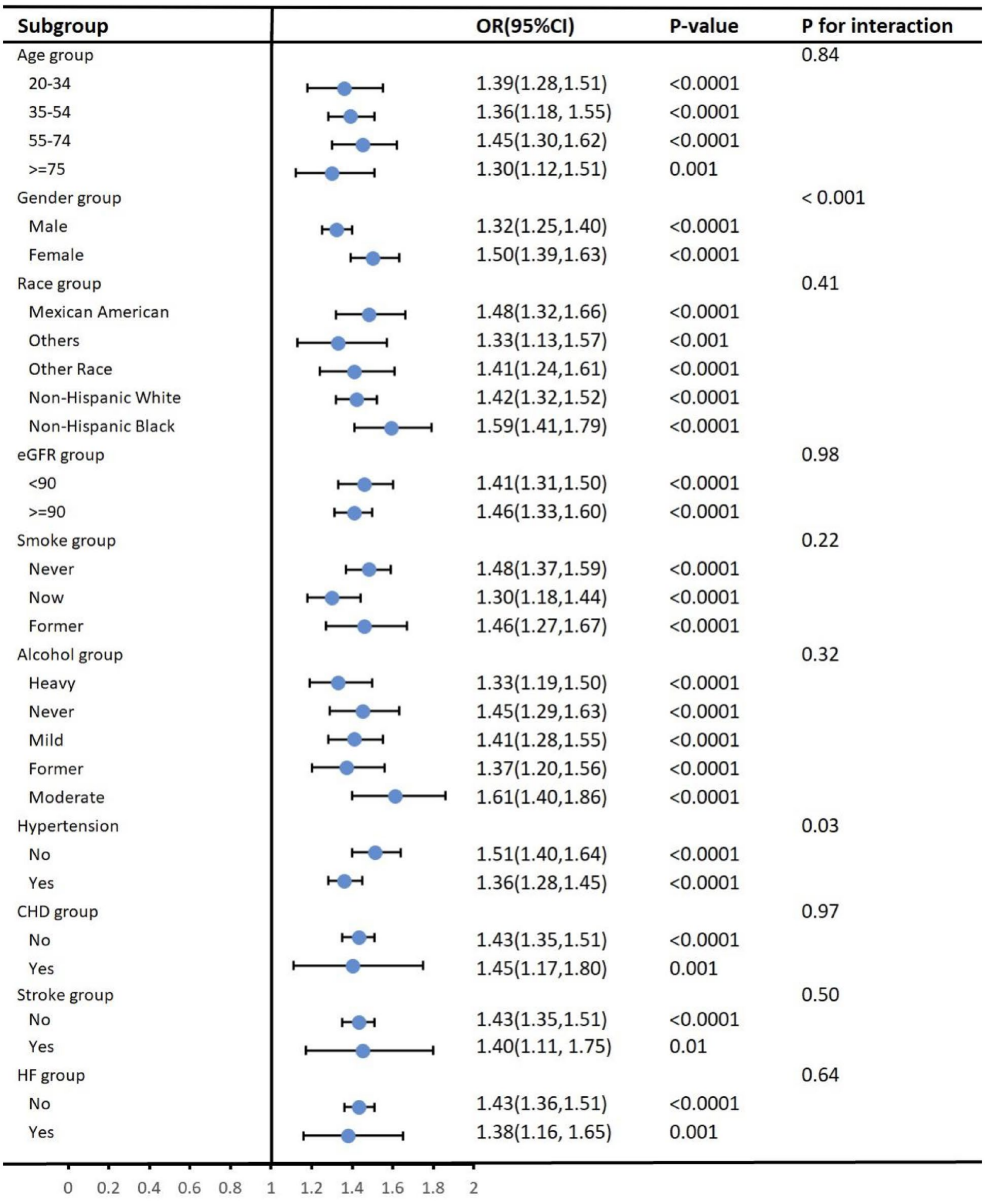
Subgroup analysis for the association between VAI and T2DM.

**Figure 4:**
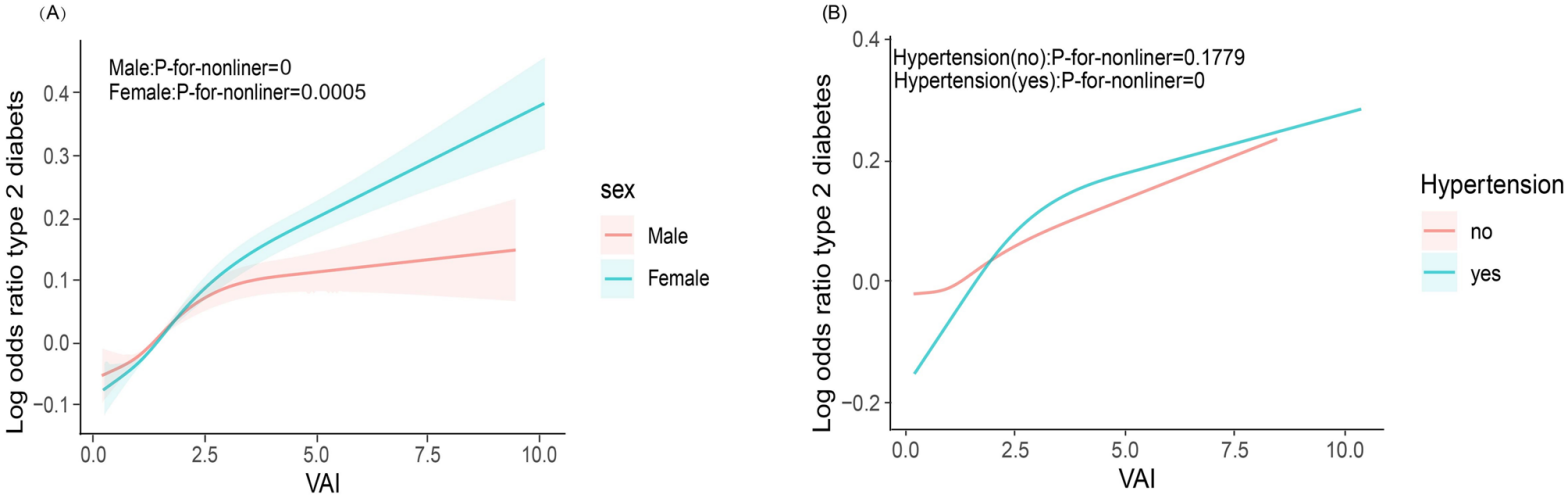
RCS analysis of gender and hypertension. (**A**) RCS analysis of gender. They adjusted for age, race, education, marital, PIR, smoke, alcohol, eGFR, TC, LDL, Alt, Ast, Physical Activity, Hypertension, Stroke, CHD, HF. (**B**) RCS analysis of hypertension. They adjusted for age, gender, race, education, marital, PIR, smoke, alcohol, eGFR, TC, LDL, Alt, Ast, Physical Activity, Stroke, CHD, HF.

Subgroup analyses, as shown in Fig. 3, showed that the prevalence between VAI and T2DM remained stable across age, race, eGFR, smoking, alcohol consumption, angina, stroke, and heart failure, and there was no interaction. We found a significant interaction in the subgroup of sex (male/female) (*P* < 0.001). In women, for every 1-unit increase in VAI, there was a 0.50-fold increase in the incidence of T2DM (OR 1.50, 95% CI 1.39–1.63). For men, each 1-unit increase in VAI was linked to a 0.32-fold increase in the odds of developing T2DM (OR 1.32, 95% CI 1.25–1.40). We also found an interaction in the subgroup of hypertensive (yes/no) (*P* = 0.03). In the hypertensive population, each 1-unit increase in VAI was accompanied by a 0.36-fold increase in the prevalence of T2DM (OR 1.36, 95% CI 1.28–1.45), whereas in the non-hypertensive population, each 1-unit increase in VAI was accompanied by a 0.51-fold increase in the prevalence of T2DM (OR 1.51, 95% CI 1.40–1.65). As shown in Fig. 4, women were more likely to develop T2DM than men (Fig. 4A) and as VAI increased nonhypertensive patients were more likely to develop T2DM than hypertensive patients (Fig. 4B).

## Discussion

In this research study that investigated the association between VAI and T2DM in adults in the US using a large 6-period cross-sectional study from the NHANES database, the main finding that VAI is positively associated with T2DM, while the non-linear association between VAI and T2DM is a secondary result. Thus, we propose that VAI can be used as a predictor of T2DM. A total of 11,214 subjects were included, and the prevalence of T2DM was 15.83%. After adjusting for confounders, each 1-unit increase of VAI was related to a 43% rise in T2DM prevalence. When VAI was considered as a categorical variable, the prevalence of T2DM was significantly higher in VAI quartile four than in quartile one. Our subgroup analysis uncovered an interaction between gender and hypertension in both subgroups and a higher prevalence in the female, non-hypertensive population than in the male, hypertensive population. The higher prevalence in women than in men is due to the role of estrogen in regulating insulin sensitivity. Estrogen acts as a protector of ectopic lipids by inhibiting the inflammatory response in white fat, which in turn inhibits oxidative stress^19,20^. With aging women have less estrogen and are more prone to IR than men. Koutsari found that women have a higher capacity to process non-oxidized free fatty acids (FFA) than men, and that non-oxidized processing of free fatty acids may increase very low-density lipoproteins (VLDL) and TG and cause insulin resistance (IR), leading to metabolic disorders^21^. In addition, it has been shown that fatty acid oxidation rates differ between genders, with women having lower levels of basal fatty acid oxidation and being more susceptible to metabolic disorders^22^. Therefore, in the future, we should pay more attention to T2DM in female, non-hypertensive population and explore it through basic experiments.

As quality of life improves and diet structures are altered, the prevalence of obesity is growing worldwide^23^. Persons with higher fat accumulation and obesity are more likely to suffer from T2DM, hypertension, and atherosclerosis, and these issues have measurable effects on the human life span^24,25^. Persons with fat concentrated near visceral organs are more susceptible to insulin resistance and T2DM than are persons in which fat deposition tends to be more subcutaneous^26,27^. Although BMI is the most frequently used method for assessing total fat deposition, it cannot differentiate between subcutaneous and visceral fat and thus cannot accurately evaluate obesity^28^. Similarly, WC, a simple and reliable measure for assessing abdominal obesity, has been used to measure total body fat^29^, but this measure, like BMI, cannot differentiate between subcutaneous and visceral fat^30^. Compared with BMI and WC, VAI can evaluate visceral fat distribution effectively and has good predictive ability, and this measure has been shown to be closely related to metabolic diseases^31,32^. VAI is a relatively simple and rapid assessment that is less costly than other tools, and it is on the cutting edge of glucose and insulin. An additional strength of VAI is that it includes both anthropometric parameters and metabolic indexes, which are useful in the evaluation of the distribution of visceral fat and functional abnormalities^33^. The VAI index has been reported to be significantly associated with metabolic syndromes including insulin resistance^34,35^, and the use of VAI has played an important role in the evaluation of hypertension, abdominal aortic calcification, and cardiovascular diseases^14,36,37^.

Diabetes mellitus is a multifactorial metabolic disease with a complex etiology involving genetic, environmental, physiological, behavioral, social, and economic factors^38^, and T2DM is the primary manifestation of this disorder. Obesity is often associated with abnormalities of lipid metabolism, and the more accumulated fat correlates with a higher the risk of T2DM in both male and female subjects. Increased adipose tissue leads to insulin resistance, reduced systemic inflammatory responses, and immune-metabolic disorders, resulting in decreased *β*-cell function and abnormally elevated blood glucose^39^. In T2DM, as adipocytes continue to increase, adipose tissue expands abnormally, leading to increased surface area, which lowers glucose transport efficiency and reduces the cellular insulin response, leading to diabetes^40^. Multiple studies have shown the utility of VAI in the clinical assessment of metabolic disorders. A cross-sectional study by Kai et al.^35^ included 27,309 adults in the United States and found that higher VAI was associated with insulin resistance(IR), and the VAI value could be used as a predictor of risk of developing IR. In another study that also included 4437 participants, Yuan et al.^7^ found that VAI was an independent risk indicator for FPG with good predictive value and could be used as a surrogate for clinical assessment of risk of hyperglycemia and could be used to guide treatment of hyperglycemia. Han et al.^41^ concluded that VAI was positively correlated with the risk of T2DM in a rural study in China. Shang et al.^42^ explored the association between VAI and the risk of T2DM in Japanese adults. Kalapur et al.^43^ found that VAI was higher in 30 DM patients than in 30 non-DM patients and thus that there was a correlation between VAI and DM. Yang et al.^44^ included 824 first-degree relatives of T2DM patients with an unknown history of abnormal glucose regulation, and the subjects had higher average VAI values than did those with normal glucose regulation. These studies and our own work have consistently shown that VAI is closely related to T2DM, and thus we believe that a large-sample cross-sectional study is warranted.

The present study was associated with several limitations. First, as the study was a cross-sectional analysis, a causal relationship between VAI and T2DM prevalence could not be determined. Second, although adjustments for confounders were made, bias due to unknown factors could not be wholly excluded. In addition, the survey data that was used in this study did not include follow-up data. Finally, due to national, special populations, and ethnic differences, further research is needed to determine whether the correlations observed in this study are influenced by ethnic factors or other differences between populations.

## Conclusion

This study demonstrated a positive association between VAI and T2DM. VAI can be used as an indicator for the clinical assessment of T2DM and has an excellent predictive value for T2DM prevalence. However, the potential mechanisms linking VAI to T2DM need to be further analyzed by large-scale prospective studies.

### Supplementary Information

Supplementary Information.

## Data Availability

Publicly available datasets were analyzed in this study. This data can be found here: https://www.cdc.gov/nchs/nhanes/.
