# Supplementary material for: The association between visceral adiposity index and risk of type 2 diabetes mellitus
Source: Sci Rep. 2024 Jul 18;14:16634. doi: 10.1038/s41598-024-67430-x (PMC11258278; doi:10.1038/s41598-024-67430-x)
Supplement: Supplementary file 1 — Supplementary Information. [file 41598_2024_67430_MOESM1_ESM.docx]

**Supplementary table S1|** Baseline information for the included participants according to Visceral Adiposity Index quartiles .

| Variable | Total  (n=11214) | Q1  (n=2803) | Q2  (n=2803) | Q3  (n=2802) | Q4  (n=2806) | P-value |
| --- | --- | --- | --- | --- | --- | --- |
| Age, years | 47.65±0.29 | 44.72±0.59 | 46.91±0.39 | 48.83±0.42 | 50.25±0.40 | < 0.0001 |
| Gender, n(%) |  |  |  |  |  | 0.15 |
| Female | 5657(50.60) | 1323(48.40) | 1420(50.84) | 1438(52.27) | 1476(51.00) |  |
| Male | 5557(49.40) | 1480(51.60) | 1383(49.16) | 1364(47.73) | 1330(49.00) |  |
| Race, n(%) |  |  |  |  |  | < 0.0001 |
| Mexican American | 1655( 8.08) | 263(5.83) | 375(7.54) | 492(9.31) | 525(9.75) |  |
| Non-Hispanic Black | 2192( 9.76) | 846(14.94) | 630(11.02) | 436( 7.90) | 280( 4.95) |  |
| Non-Hispanic White | 4966(69.69) | 1122(67.04) | 1213(69.53) | 1243(69.26) | 1388(72.99) |  |
| Other Race | 1252( 7.11) | 360(7.69) | 300(6.25) | 315(7.86) | 277(6.63) |  |
| Others | 1149( 5.37) | 212(4.50) | 285(5.66) | 316(5.67) | 336(5.68) |  |
| Education, n(%) |  |  |  |  |  | < 0.0001 |
| 9-11th grade | 1553(10.20) | 335( 7.79) | 352( 9.34) | 411(11.29) | 455(12.48) |  |
| College graduate or above | 2791(31.56) | 905(40.84) | 749(31.91) | 630(28.11) | 507(24.97) |  |
| High school graduate | 2545(22.60) | 559(19.17) | 654(23.50) | 649(22.37) | 683(25.48) |  |
| Less than 9th grade | 1015( 4.63) | 156(3.15) | 226(4.17) | 281(4.89) | 352(6.35) |  |
| Some College | 3310(31.02) | 848(29.04) | 822(31.09) | 831(33.34) | 809(30.73) |  |
| Marital status, n(%) |  |  |  |  |  | < 0.0001 |
| Divorced | 1253(10.61) | 268( 8.69) | 326(11.18) | 306(10.86) | 353(11.79) |  |
| Living with partner | 917( 8.08) | 252(9.00) | 229(7.84) | 213(8.10) | 223(7.35) |  |
| Married | 5831(56.23) | 1339(54.25) | 1434(54.84) | 1518(56.41) | 1540(59.48) |  |
| Never married | 2018(17.68) | 694(22.29) | 535(19.02) | 451(17.20) | 338(12.05) |  |
| Separated | 371( 2.12) | 85(1.64) | 91(2.44) | 95(2.02) | 100(2.39) |  |
| Widowed | 824( 5.28) | 165(4.12) | 188(4.68) | 219(5.42) | 252(6.94) |  |
| PIR |  |  |  |  |  | < 0.0001 |
| <1.30 | 3450(20.75) | 741(17.68) | 803(19.82) | 879(21.25) | 1027(24.34) |  |
| 1.30-3.50 | 4262(35.48) | 1073(34.20) | 1069(34.90) | 1083(37.34) | 1037(35.58) |  |
| >=3.50 | 3502(43.77) | 989(48.12) | 931(45.27) | 840(41.41) | 742(40.08) |  |
| eGFR, mL/min/1.73 m2 | 94.92±0.37 | 98.52±0.68 | 95.51±0.52 | 93.54±0.54 | 91.95±0.61 | < 0.0001 |
| BMI, kg/m2 | 28.97±0.11 | 25.78±0.15 | 28.21±0.18 | 30.17±0.17 | 31.86±0.16 | < 0.0001 |
| WC, cm | 99.36±0.27 | 90.36±0.36 | 97.09±0.46 | 102.70±0.37 | 107.66±0.42 | < 0.0001 |
| FPG, mmol/L | 5.90±0.02 | 5.53±0.03 | 5.67±0.03 | 5.96±0.04 | 6.46±0.06 | < 0.0001 |
| HbA1c, % | 5.62±0.01 | 5.41±0.02 | 5.51±0.01 | 5.67±0.02 | 5.91±0.04 | < 0.0001 |
| Physical Activity,METs | 3853.99±93.58 | 4761.67±191.08 | 3857.54±122.79 | 3531.08±148.04 | 3228.47±139.02 | < 0.0001 |
| TC, mmol/L | 4.98±0.02 | 4.75±0.03 | 4.91±0.02 | 4.99±0.03 | 5.29±0.03 | < 0.0001 |
| TG, mmol/L | 1.37±0.01 | 0.68±0.01 | 1.02±0.01 | 1.40±0.01 | 2.39±0.02 | < 0.0001 |
| HDL, mmol/L | 1.41±0.01 | 1.77±0.01 | 1.48±0.01 | 1.29±0.01 | 1.08±0.01 | < 0.0001 |
| LDL, mmol/L | 2.95±0.01 | 2.64±0.02 | 2.96±0.02 | 3.07±0.02 | 3.15±0.02 | < 0.0001 |
| Alt, U/L | 25.10±0.21 | 22.39±0.49 | 23.88±0.41 | 25.26±0.32 | 28.94±0.40 | < 0.0001 |
| Ast, U/L | 25.08±0.19 | 25.10±0.42 | 24.59±0.35 | 24.45±0.29 | 26.17±0.44 | 0.01 |
| VAI | 1.80±0.02 | 0.59±0.00 | 1.10±0.00 | 1.79±0.01 | 3.74±0.04 | < 0.0001 |
| Smoking status, n(%) |  |  |  |  |  | < 0.0001 |
| Former | 2773(25.70) | 622(23.68) | 665(24.38) | 727(27.05) | 759(27.79) |  |
| Never | 6179(55.28) | 1708(61.32) | 1605(57.89) | 1509(52.83) | 1357(48.82) |  |
| Now | 2262(19.02) | 473(14.99) | 533(17.73) | 566(20.11) | 690(23.39) |  |
| Alcohol use, n(%) |  |  |  |  |  | < 0.0001 |
| Former | 1713(12.51) | 321( 8.82) | 369(11.01) | 464(13.63) | 559(16.71) |  |
| Heavy | 2293(21.05) | 543(19.76) | 593(22.58) | 594(21.91) | 563(20.01) |  |
| Mild | 3958(38.46) | 1089(41.34) | 1020(38.30) | 944(36.47) | 905(37.58) |  |
| Moderate | 1757(17.83) | 515(21.00) | 446(17.22) | 414(17.64) | 382(15.36) |  |
| Never | 1493(10.15) | 335( 9.07) | 375(10.89) | 386(10.34) | 397(10.34) |  |
| Hypertension, n(%) |  |  |  |  |  | < 0.0001 |
| No | 6492(62.23) | 1901(74.31) | 1693(65.29) | 1526(58.64) | 1372(50.22) |  |
| Yes | 4722(37.77) | 902(25.69) | 1110(34.71) | 1276(41.36) | 1434(49.78) |  |
| Stroke, n(%) |  |  |  |  |  | 0.08 |
| No | 10785(97.10) | 2721(97.89) | 2693(97.19) | 2697(96.87) | 2674(96.43) |  |
| Yes | 429( 2.90) | 82(2.11) | 110(2.81) | 105(3.13) | 132(3.57) |  |
| CHD, n(%) |  |  |  |  |  | < 0.001 |
| No | 10766(96.52) | 2720(97.31) | 2701(97.12) | 2688(96.73) | 2657(94.91) |  |
| Yes | 448( 3.48) | 83(2.69) | 102(2.88) | 114(3.27) | 149(5.09) |  |
| HF, n(%) |  |  |  |  |  | < 0.0001 |
| No | 10889(97.74) | 2752(98.77) | 2732(98.24) | 2725(97.93) | 2680(96.00) |  |
| Yes | 325( 2.26) | 51(1.23) | 71(1.76) | 77(2.07) | 126(4.00) |  |
| T2DM, n(%) |  |  |  |  |  | < 0.0001 |
| No | 8881(84.17) | 2490(92.67) | 2367(89.39) | 2130(81.66) | 1894(72.65) |  |
| Yes | 2333(15.83) | 313( 7.33) | 436(10.61) | 672(18.34) | 912(27.35) |  |

Abbreviations: quartile of Visceral Adiposity Index (VAI), Q1(≤0.849), Q2(0.849<VAI≤1.379), Q3(1.379<VAI≤2.324), Q4(>2.324); Income to poverty ratio(PIR),eGFR, estimated glomerular filtration rate; BMI, body mass index; WC, waist circumference; FPG, fasting plasma glucose; HbA1c, hemoglobin A1c; TC, total cholesterol; TG, triglyceride; HDL, high-density lipoprotein; LDL-c, low-density lipoprotein-cholesterol; ALT, alanine aminotransferase; AST, aspartate aminotransferase; CHD, coronary heart disease; HF, heart failure; DM, diabetes mellitus.
